# How and Why DNA Barcodes Underestimate the Diversity of Microbial Eukaryotes

**DOI:** 10.1371/journal.pone.0016342

**Published:** 2011-02-10

**Authors:** Gwenael Piganeau, Adam Eyre-Walker, Nigel Grimsley, Hervé Moreau

**Affiliations:** 1 UPMC Univ Paris 06, UMR 7232, Observatoire Océanologique, Banyuls-sur-Mer, France; 2 CNRS, UMR 7232, Observatoire Océanologique, Banyuls-sur-Mer, France; 3 School of Life Sciences, Sussex University, Brighton, United Kingdom; Université Paris Sud, France

## Abstract

**Background:**

Because many picoplanktonic eukaryotic species cannot currently be maintained in culture, direct sequencing of PCR-amplified 18S ribosomal gene DNA fragments from filtered sea-water has been successfully used to investigate the astounding diversity of these organisms. The recognition of many novel planktonic organisms is thus based solely on their 18S rDNA sequence. However, a species delimited by its 18S rDNA sequence might contain many cryptic species, which are highly differentiated in their protein coding sequences.

**Principal Findings:**

Here, we investigate the issue of species identification from one gene to the whole genome sequence. Using 52 whole genome DNA sequences, we estimated the global genetic divergence in protein coding genes between organisms from different lineages and compared this to their ribosomal gene sequence divergences. We show that this relationship between proteome divergence and 18S divergence is lineage dependant. Unicellular lineages have especially low 18S divergences relative to their protein sequence divergences, suggesting that 18S ribosomal genes are too conservative to assess planktonic eukaryotic diversity. We provide an explanation for this lineage dependency, which suggests that most species with large effective population sizes will show far less divergence in 18S than protein coding sequences.

**Conclusions:**

There is therefore a trade-off between using genes that are easy to amplify in all species, but which by their nature are highly conserved and underestimate the true number of species, and using genes that give a better description of the number of species, but which are more difficult to amplify. We have shown that this trade-off differs between unicellular and multicellular organisms as a likely consequence of differences in effective population sizes. We anticipate that biodiversity of microbial eukaryotic species is underestimated and that numerous “cryptic species” will become discernable with the future acquisition of genomic and metagenomic sequences.

## Introduction

Our understanding of the evolution of eukaryotes was revolutionized when it became possible to compare sequenced marker genes, notably the ribosomal genes, among many organisms [1]. In practice, ribosomal genes are often the only markers available for estimating the diversity of unicellular eukaryotes, especially in the Chromalveolates, Excavata and Rhizaria group which have few sequenced representatives. They are also the only markers used in the analysis of environmental or metagenomic DNA sequence datasets [2], [3]. It is thus becoming crucially important to know how well these signatures represent the extent of diversity in the exploding body of data that will become available over the next ten years as revolutionary sequencing technology are used in panoceanic metagenomic campaigns [4], [5]. Marine metagenomics studies rely on a pragmatic species concept; sequences are declared as being from separate species or genera based upon an arbitrary level of sequence divergence at a marker locus, typically the 18S rDNA ribosomal gene [6]. In this study, we analysed how genome divergence, estimated from amino-acid changes in protein coding genes, compares with 18S ribosomal divergence, the universal marker for planktonic eukaryotes biodiversity.

## Methods

Whole genome predicted proteins data was downloaded from GenBank, JGI, Genolevure, Ensembl [7], PLAZA [8] and organisms' dedicated databases (Table 1). Complete 18S rDNA sequences were downloaded from GenBank or extracted from the whole genome sequence by screening the complete genome with complete 18S rDNA sequence from a closely related species. For the primate data, 18S rDNA sequenced were reassembled from the GenBank Trace archive (Table 1).

**Table 1:** Genome data and 18S rDNA data used for analysis.

Species	Database	URL	Release	Gene	18S rDNA sequence
**DIPTERA**					
*Aedes aegypti*	VectorBase	http://aaegypti.vectorbase.org/	AaegL1.1	16789	from genome assembly
*Culex pipiens*	VectorBase	http://cpipiens.vectorbase.org/	CpipJ1.2	18883	from genome assembly
*Drosophila ananassae*	flybase	ftp://ftp.flybase.net/genomes/	r1.3	15070	from genome assembly
*Drosophila melanogaster*	flybase	ftp://ftp.flybase.net/genomes/	r5.9	21064	M21017.1
*Drosophila erecta*	flybase	ftp://ftp.flybase.net/genomes/	r1.3	15048	from genome assembly
*Drosophila yakuba*	flybase	ftp://ftp.flybase.net/genomes/	r1.3	16082	from genome assembly
*Drosophila grimshawi*	flybase	ftp://ftp.flybase.net/genomes/	r1.3	14986	from genome assembly
*Drosophila willistoni*	flybase	ftp://ftp.flybase.net/genomes/	r1.3	15513	from genome assembly
*Drosophila persimilis*	flybase	ftp://ftp.flybase.net/genomes/	r1.3	16878	from genome assembly
*Drosophila pseudoobscura*	flybase	ftp://ftp.flybase.net/genomes/	r2.3	16071	AY03717
*Drosophila sechellia*	flybase	ftp://ftp.flybase.net/genomes/	r1.3	16471	from genome assembly
*Drosophila simulans*	flybase	ftp://ftp.flybase.net/genomes/	r1.3	15415	AY037174.1
**VERTEBRATA**					
*Homo sapiens*	Ensembl	http://archive.ensembl.org/	v54	47509	M10098
*Pan troglodytes*	Ensembl	http://archive.ensembl.org/	v54	34142	rebuilt from Trace
*Mus musculus*	Ensembl	http://archive.ensembl.org/	v38	31986	X00686.1
*Rattus norvegicus*	Ensembl	http://archive.ensembl.org/	v54	32948	X01117
*Macaca Mulatta*	Ensembl	http://archive.ensembl.org/	v54	36384	rebuilt from Trace
*Pongo pygmaeus*	Ensembl	http://archive.ensembl.org/	v54	23533	rebuilt from Trace
*Bos Taurus*	Ensembl	http://archive.ensembl.org/	v54	26977	DQ222453.1
*Equus caballus*	Ensembl	http://archive.ensembl.org/	v54	22641	AJ311673.1
*Gallus gallus*	Ensembl	http://archive.ensembl.org/	v47	22195	AF173612
*Xenopus tropicalis*	Ensembl	http://archive.ensembl.org/	v54	27710	from genome assembly
**STREPTOPHYTA**					
*Oryza sativa*	Rice	http://rice.plantbiology.msu.edu/	v6	67393	from genome assembly
*Sorghum bicolor*	JGI	http://genome.jgi-psf.org/Sorbi1/Sorbi1.download.ftp.html	Sbi1_4	34496	from genome assembly
*Populus trichocharpa*	JGI	http://genome.jgi-psf.org/	v1.1	45555	from genome assembly
*Medicago truncatula*	Medicago	http://www.medicago.org/		44830	AF093506.1
*Arabidopsis thaliana*	TAIR	http://www.arabidopsis.org/index.jsp		27855	X16077.1
*Arabidopsis lyrata*	JGI	http://www.jgi.doe.gov/genome-projects/		32670	from genome assembly
*Carica papaya*	Carica	asgpb.mhpcc.hawaii.edu/papaya/		24782	from genome assembly
*Vitis vinifera*	Genoscope	http://www.genoscope.cns.fr/		30434	from genome assembly
**CHLOROPHYTA**					
*Micromonas pusilla CCMP1545*	JGI	http://www.jgi.doe.gov/genome-projects/	V2	10242	from genome assembly
*Micromonas pusilla RCC299*	JGI	http://www.jgi.doe.gov/genome-projects/	V3	10109	from genome assembly
*Ostreococcus lucimarinus*	JGI	http://www.jgi.doe.gov/genome-projects/	v2	7651	from genome assembly
*Ostreococcus RCC809*	JGI	http://www.jgi.doe.gov/genome-projects/	v1	7773	from genome assembly
*Bathycoccus prasinos*	Genoscope	http://bioinformatics.psb.ugent.be/	V1	8747	from genome assembly
*Ostreococcus tauri*	Bogas	http://bioinformatics.psb.ugent.be/	v2	7725	from genome assembly
**SACCHAROMYCETACEAE**					
*Saccharomyces cerevisiae*	SGD	http://www.yeastgenome.org/		5914	Z75578
*Saccharomyces paradoxus*	MIT	http://www.broad.mit.edu/annotation/		4774	X97806
*Saccharomyces mikatae*	Broad	http://fungal.genome.duke.edu/		5884	AB040998
Saccharomyces kudriavzevi	WUSTL	http://fungal.genome.duke.edu/		6371	AACI02000378.1
*Saccharomyces bayanus*	MIT	http://www.broad.mit.edu/annotation/		4492	X97777
*Saccharomyces castellii*	WUSTL	http://fungal.genome.duke.edu/		5864	AACF01000230.1
*Lachancea waltii*	Genolevure	http://fungal.genome.duke.edu/		5350	AADM01000401.1
*Lachancea thermotolerans*	Genolevure	http://fungal.genome.duke.edu/		5092	X89526.1

Twenty six phylogenetic independent comparisons were inferred from couple of species with less than 5% 18S rDNA divergences (all species pairs, number of genes and phylogenies within each lineage are available in Figure S1).

All orthologous gene pairs between species were inferred by reciprocal best hit (e-value 10^−3^). We retrieved the common set of orthologous genes within each lineage by extracting the orthologous genes present in all pairwise species comparisons. We thus obtained 2151 common gene pairs in Chlorophyta, 5051 in Diptera, 2925 in Saccharomyceta, 4160 in Streptophyta and 5949 in Vertebrata. Protein sequences were aligned with the Needleman Wunsch algorithm [9] and processed with custom C codes to compute amino-acid identities over the concatenated alignments. Substitution rates d_AA_ were estimated via maximum likelihood with the PAML package (Jones [10] substitution matrix) [11].

We manually inspected multiple sequence alignments to identify common sites of the 18S rDNA : large insertions occurring in some sequences were excluded from the alignment to get consistent divergence estimate across pairwise comparions. All 18S rDNA pairs were aligned with the Needleman Wunsch algorithm to estimate pairwise differences, The nucleotide substitution rates of the 18S rDNA were estimates with the PAML package (HKY85 substitution model).

Statistical analyses were performed with the R software.

## Results

### The rate of 18S rDNA and protein evolution

**Figure 1:**
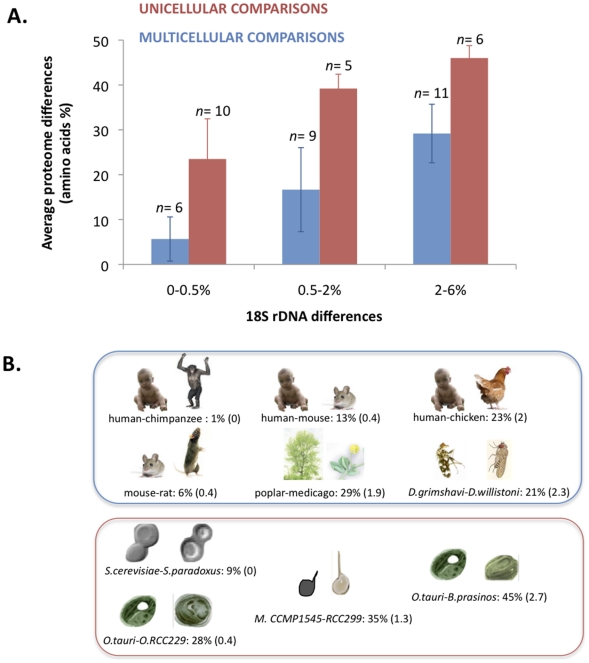
18S rDNA versus proteome divergence in unicellular and multicellular lineages. A. Average proteome (amino-acid) and 18S rDNA differences (%) for 21 unicellular and 26 multicellular pairwise comparisons. The first class of 18S rDNA sequence differences limit, 0.5%, is the smallest threshold used to delineate Operational Taxonomic Units (OTU) in planktonic eukaryotes [26]. B. Selected examples of pairwise comparisons in each 18S rDNA divergence class: percent of amino-acid divergence (percent of 18S rDNA differences).

**Figure 2:**
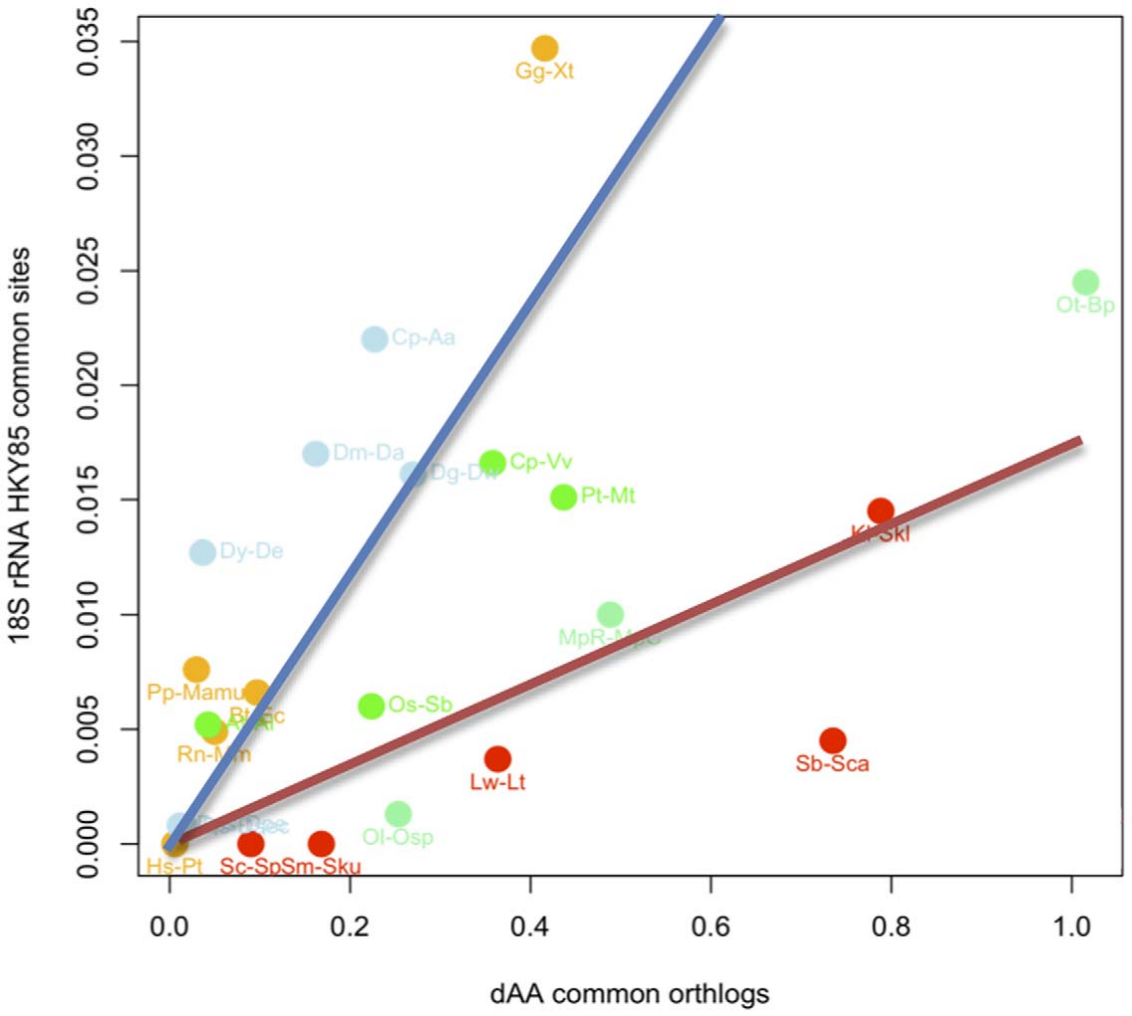
18s rDNA evolution rates versus Amino-acid evolution rates for all common orthologous genes within lineages for independent pairs of species. Yellow: Vertebrates, Green: Streptophytes, Light blue: Diptera, Light green: Chlorophyta, Red: Saccharomyceta.

Recent genome and metagenomic projects have highlighted the surprising discrepancy between 18S rDNA divergence and whole genome divergence in some phytoplanktonic species [12], [13], [14], [15], that are keystone players in the global carbon cycling [16]. Here we investigated the generality of this observation among both unicellular and muticellular eukaryotes. We compared the 18S rDNA and the proteome divergence across all available eukaryotic genomes in 2 unicellular (Baker's yeast and green alga) and 3 multicellular lineages (Vertebrates, Diptera and Land plants). We found that for a given level of rDNA divergence, unicellular eukaryotes had substantially greater proteome divergence than multicellular eukaryotes (Figure 1A). This can be more formally tested using an analysis of covariance of proteome versus rDNA divergence, forcing the regression lines through the origin and testing for equality of slopes : the test is highly significantly different (p<0.0001) (Figure 1A). Identical 18S rDNA sequences between two unicellular species may correspond to proteome divergences of the same order as those observed between Xenopus and Chicken or the Poplar tree and the grass Medicago (Figure 1B). Amino-acid divergences between orthologous genes are only one of the many hallmarks of evolutionary divergence after speciation. A genomic species definition for protists based on proteome divergence is stringent, because genomic rearrangements, the acquisition of new genes via duplication or even a few mutations within a subset of genes may be sufficient to delineate two species [17], [18]. To reduce possible effects of amino-acid content, base composition and non-independency of observations, we computed the substitution rates on a common set of orthologs within each lineage across all independent pairwise comparisons. Consistent with the raw number of difference estimates, the evolution rate of the 18S rDNA relative to the proteome is much lower in unicellular species (analysis of covariance unicellulars versus multicellulars *p* = 0.048) (Figure 2).

## Discussion

### A population genetic explanation

What could be the cause of this decoupling between 18S rDNA and proteome divergence in unicellar versus multicellular species? There are two general explanations; first, the proportion of mutations that are strongly deleterious is higher in 18S rDNA, when compared to protein sequences, in unicells compared to multicells. One could argue that the 18S rDNA may be under much more stronger selection in unicells, where fitness may depend more directly from transcription efficiency than in multicellular species. Second, the rate of adaptive evolution could be higher in protein sequences in unicells compared to multicells. It is difficult to differentiate between these possibilities. However, unicells and multicells are likely to differ in their effective population sizes and this suggests a simple explanation; that the proportion of effectively neutral mutations changes more in response to differences in the effective population size in the 18S rDNA than in the proteome. This can be formalised as follows. Let us assume that all mutations are deleterious (or effectively neutral) and that the distribution of fitness effects is a gamma distribution. Under a gamma distribution it can be shown that the rate of evolution, *R*, is a function of the mutation rate, μ, divergence time, *t*, and the Distribution of Fitness effects of new mutations, fully described by the shape parameters, *ß*, and the effective population size, *Ne* [19], [20], [21].
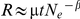
We can thus express the relative ratio between the rate of evolution of the 18S rDNA, *Rr*, and the rate of evolution of the proteome, *Rp*, in one lineage as a function of three parameters, where *N_e_* is the average effective population size within a lineage:
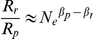
This ratio can be estimated from our observations (Figure 2) by taking the linear regression coefficient for each lineage (slope = 0.017 for unicellulars and slope = 0.059 for multicellular organims).

If we assume that unicells have an effective population size, *Ne*, that is 1000 to 1,000,000 times larger than in multicells, then *ß_r_*−*ß_p_* would be between −0.2 and −0.1 to explain the differences in the regression slopes. So quite modest differences in the distribution of fitness effects, and effective population sizes can lead to substantial differences in the relative rates at which the 18S rDNA and protein coding sequences evolve. Recent estimates of *ß*_p_ for nuclear genes in Humans and Drosophila are 0.2 and 0.35 respectively [22] [23]and we thus expect *ß*_r_ to take values smaller than 0.25.

Large effective population sizes of unicellular eukaryotes may thus provide an explanation for the surprising low divergence of 18S rDNA relative to the genome divergence. More generally, this conclusion applies to any barcoding gene sufficiently constrained to provide a large phylogenetic spread over the eukaryotic tree of life, suggesting that biodiversity studies have to make a trade-off between phylogenetic spread and phylogenetic depth for a given barcoding gene. Given the present diversity estimates of eukaryotic unicells from conserved barcoding genes like the 18S rDNA [24], [25], we thus anticipate that future eukaryotic planktonic metagenomic and genomic analysis will lead to an increase in the number of species.

## Supporting Information

Figure S1 Phylogenetic relationships and number of genes used for independent comparison.
(TIFF)
