# Correction: How and Why DNA Barcodes Underestimate the Diversity of Microbial Eukaryotes

**DOI:** 10.1371/annotation/c12aac06-71d2-4749-91de-46c458e7a4eb

**Published:** 2012-04-03

**Authors:** Gwenael Piganeau, Adam Eyre-Walker, Nigel Grimsley, Hervé Moreau

There was an error in the author byline. Severine Jancek was excluded. The correct byline is: Gwenael Piganeau, Adam Eyre-Walker, Severine Jancek, Nigel Grimsley, Herve Moreau.

Severine Jancek's affiliation is 4 Institut de Recherche sur la Biologie de l'Insecte, UMR 6035, CNRS, Université François Rabelais, 37200 Tours, France.

The correct citation is: Piganeau G, Eyre-Walker A, Jancek S, Grimsley N, et al. (2011) How and Why DNA Barcodes Underestimate the Diversity of Microbial Eukaryotes. PLoS ONE 6(2): e16342. doi:10.1371/journal.pone.0016342

Severine Jancek's author contribution is: Analyzed the data. 

