# Supplementary figures and images for: How and Why DNA Barcodes Underestimate the Diversity of Microbial Eukaryotes
Source: PLoS One. 2011 Feb 10;6(2):e16342. doi: 10.1371/journal.pone.0016342 (PMC3037371; doi:10.1371/journal.pone.0016342)

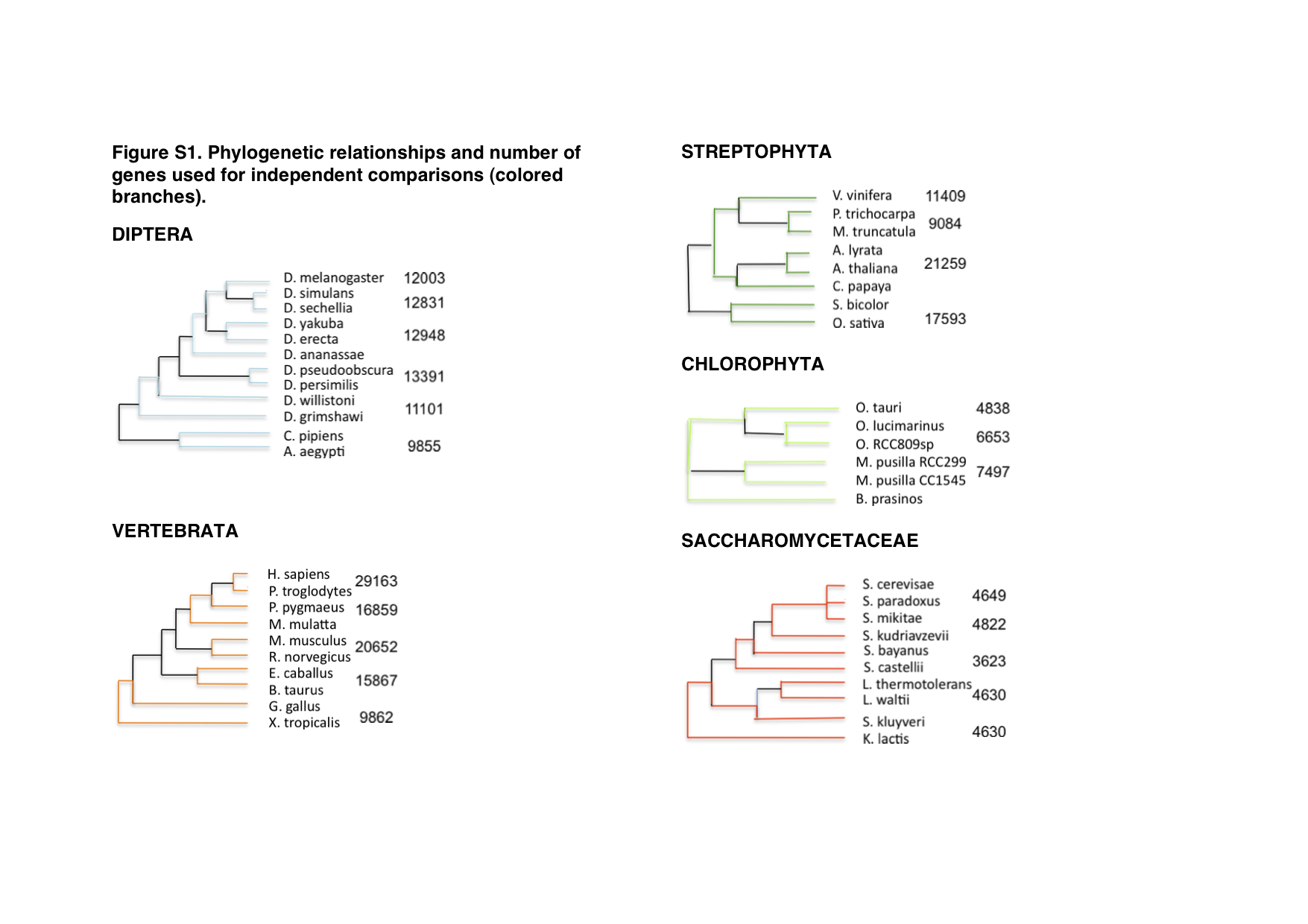

Supplement: Figure S1 — Phylogenetic relationships and number of genes used for independent comparison. (TIFF) [file pone.0016342.s001.tiff]
